# A retrospective study on the treatment of superficial infantile hemangiomas of the head and neck using topical compresses with 0.5% timolol maleate eye drops

**DOI:** 10.3389/fped.2025.1734987

**Published:** 2026-01-19

**Authors:** Li Yang, Feifan Chen, Kunpeng Li, Jiajun Chen, Wenying Liu, Yi Ji, Meng Xia, Jing Xie, Ke Ding, Qiang Zeng, Fang Hou

**Affiliations:** 1 Department of Pediatric Surgery, Sichuan Provincial People’s Hospital, School of Medicine, University of Electronic Science and Technology of China, Chengdu, China; 2 North Sichuan Medical College, Nanchong, China; 3 Southwest Medical University, Luzhou, China; 4 Department of Pediatric Surgery, West China Second University Hospital, Sichuan University, Chengdu, China; 5 Division of Oncology, Department of Pediatric Surgery, West China Hospital of Sichuan University, Chengdu, China; 6 Sichuan Provincial People's Hospital East Sichuan Hospital & Dazhou First People's Hospital, Dazhou, China

**Keywords:** beta-blocker, head and neck, infantile hemangioma, retrospective study, timolol maleate, topical treatment

## Abstract

**Introduction:**

Infantile hemangiomas, the commonest benign vascular tumors of infancy, often cluster on the head and neck where early treatment can avert permanent disfigurement, prompting us to evaluate 0.5% timolol maleate wet compresses as a non-invasive alternative to oral propranolol.

**Methods:**

We conducted a study of 359 consecutive infants treated at Sichuan Provincial People's Hospital between December 2018 and January 2024. Baseline demographics, lesion site and size, age at initial treatment, treatment duration, and follow-up period were recorded. Treatment outcomes were graded as excellent (complete regression), good (≥50% shrinkage), fair (<50% shrinkage), or poor (no change/growth). Eexcellent/good outcomes were defined as a positive therapeutic effect, while fair/poor outcomes were classified as negative therapeutic effect.

**Results:**

267 infants (74.37%) achieved positive therapeutic effect, with 117 excellent and 150 good, whereas 92 infants (25.63%) achieved negative therapeutic effect including 53 were fair and 39 poor. Treatment outcomes were significantly better when therapy began before 3 months (early age) (*U* = 9954, *Z* = − 3.256, *P* = 0.001) and for lesions ≤3 cm diameter (small size) (*U* = 2,869.5, *Z* = − 13.952, *P* < 0.001), and multivariate analysis confirmed early age (OR = 0.784, *P* = 0.024) and small size (OR = 0.113, *P* < 0.001) as independent predictors of positive therapeutic effect. Adverse events were mild: 24 (6.69%) local irritation, 41 (11.42%) transient systemic symptoms. Skin sequelae were observed in 12 (3.34%) cases.

**Discussion:**

Topical timolol compresses offer a safe, effective first-line option for superficial head-and-neck infantile hemangiomas, especially when started early and directed at smaller lesions.

## 1 Introduction

Infantile hemangioma (IH) is one of the most common benign tumors in infants and young children, with a global incidence rate of approximately 4% to 10%. And its prevalence varies depending on region, ethnicity, and research methodology (1–3). IH has the highest incidence among Caucasians in North America and Europe, with significantly increased rates also observed in premature infants, low birth weight infants, and female patients (2). IH can occur anywhere on the body, but is most commonly found on the head and neck (4). IH usually manifests within weeks of birth, with early signs including telangiectasia or erythema, typical features depend on lesion depth. Superficial dermal lesions (superficial hemangiomas) present as well-defined bright red elevations (5). Most IH experience their fastest growth between 5 and 8 weeks of age (6), reaching approximately 80% of their final size by around 3.2 months after birth (7), and reaches its maximum volume near the end of the proliferation phase (around 6 months of age).

The natural remission process of IH typically lasts 3–9 years, with most complete remission by around age 4 (8, 9), but 69% of cases develop permanent skin changes (telangiectasia, fibrofatty residues, etc.) (8, 10). Moreover, the incidence of residual skin changes is significantly higher in superficial hemangiomas (74%) than in deep hemangiomas (25%) (8). Notably, IH ulcers predominantly occur during the proliferative phase (median age of 4 months), which is closely associated with abnormal local blood supply caused by rapid tumor growth (11). While small IH may resolve spontaneously, intervention is needed for complications or head/neck lesions to avoid cosmetic/functional impairment. The timing of IH treatment is closely linked to cosmetic outcomes, the earlier intervention begins, the more favorable the prognosis. The core objectives of clinical treatment are: effectively suppressing further IH proliferation, promoting early entry into the regression phase, maximizing prevention of permanent skin sequelae (12).

Currently, the oral propranolol has become the primary first-line medication for treating IH. However, it may carries serious adverse drug reactions, requiring close monitoring when administered for the first time (13). Timolol maleate eye drops are also a non-selective potent beta-blocker initially developed for treating glaucoma (14). Subsequent literature reports indicate its significant efficacy in treating IH when applied topically, commonly available concentrations include 0.25% and 0.5%, with the 0.5% concentration being the standard for IH treatment (15). Studies indicate that topical application of timolol may exhibit efficacy comparable to propranolol for superficial IH, while causing fewer adverse reactions (16, 17), making it preferable for mild/moderate superficial cases. However, caution is needed for ulcerative IH or premature infants due to systemic absorption risks (18).

In recent years, topical timolol has demonstrated significant efficacy in the treatment of IH, emerging as a new clinical option. However, clinical consensus lacks on its treatment duration, initiatal time, dosage, frequency, applicable tumor size, and long-term safety for IH (19). Therefore, the main objective of this retrospective study was to systematically evaluated the clinical efficacy and adverse reactions of topical timolol treatment in pediatric patients with superficial IH of the head and neck across different age groups.

## 2 Data and methods

### 2.1 Clinical data

This clinical study was reviewed and approved by the Institutional Review Board of Sichuan Provincial People's Hospital, University of Electronic Science and Technology of China. Patients with superficial IH of the head and neck who received only topical treatment with timolol maleate eye drops at the Pediatric Surgery Department of Sichuan Provincial People's Hospital from December 2018 to January 2024 were selected. The diagnosis of IH was according to the International Society for the Study of Vascular Anomalies classification (ISSVA 2025) (20). Exclusion criteria include a history of allergy to beta-blocker medications, electrocardiogram findings of sinus bradycardia, prolonged P-R interval and atrioventricular block, a history of bronchial asthma, unsuitability for topical medication application near the eyes, mouth and nose, prior and concurrent treatment with other modalities, initiation of treatment at age over 12 months or under 1 month, and follow-up duration less than 12 months. Collect general information and clinical characteristics of pediatric patients. Patients with multiple IH were enrolled based on the largest and most typical tumor. For the age at which patients first received treatment, children with IH were divided into two groups based on their age at the time of initial treatment: <3 months (early age) and ≥3 months (late age). Based on the maximum diameter of IH, the tumors were classified into the small size group (0.3 cm ≤ maximum diameter ≤ 3 cm) and the large size group (3 cm < maximum diameter < 9 cm).

### 2.2 Topical timolol treatment procedure

#### 2.2.1 Method of administration

**Figure 1:**
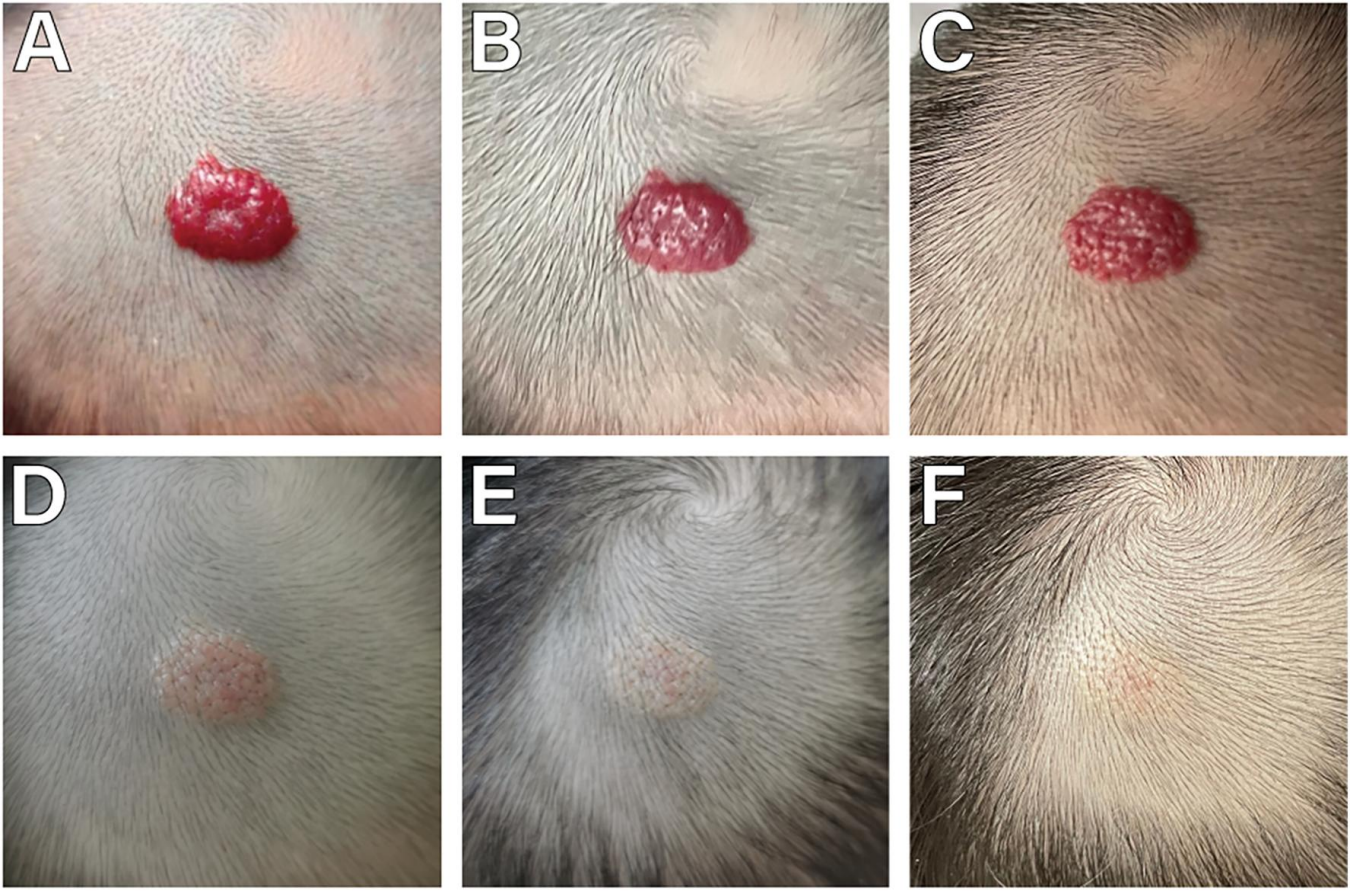
A female with a small superficial IH on the top of the head. **(A)** Before starting topical timolol therapy, at the age of 3 months and 1 week; **(B)** 1 week after starting topical timolol therapy, the IH gradually became less red; **(C)** 10 weeks after the initiation of timolol treatment, compared with the 1 week of treatment was not dramaticly; **(D)** 3 months after the initiation of timolol treatment, the IH shrinked and less red significantly; **(E)** 6 and a half months after the initiation of timolol treatment, the IH became more flatter and paled; **(F)** 10 months after the initiation of timolol treatment, the IH were almost invisible, a stepwise withdrawal of the drug was initiated.

**Figure 2:**
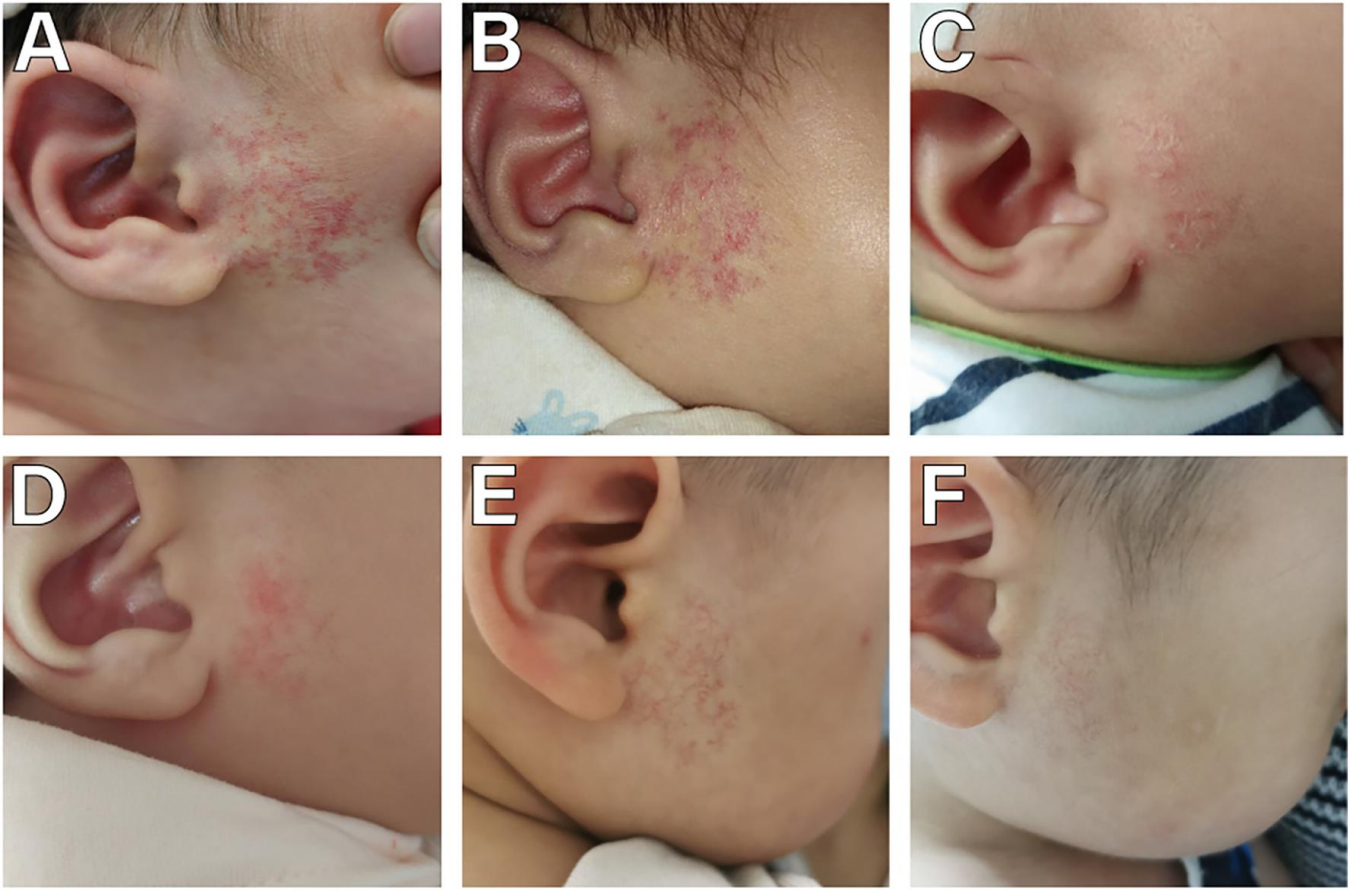
A male with a superficial IH in front of the right ear. **(A)** Before starting topical timolol therapy, at the age of 1 months; **(B)** 1 week after starting topical timolol therapy, there was no significant change in IH; **(C)** 2 months after the initiation of timolol treatment, the IH shrinked and less red, but desquamation can be observed; **(D)** 3 months after the initiation of timolol treatment, the IH became more smaller; **(E)** 10 months after the initiation of timolol treatment, the IH was reduced to less than half of its original size; **(F)** 25 months after the timolol treatment stopped, no skin sequelae was observed.

**Figure 3:**
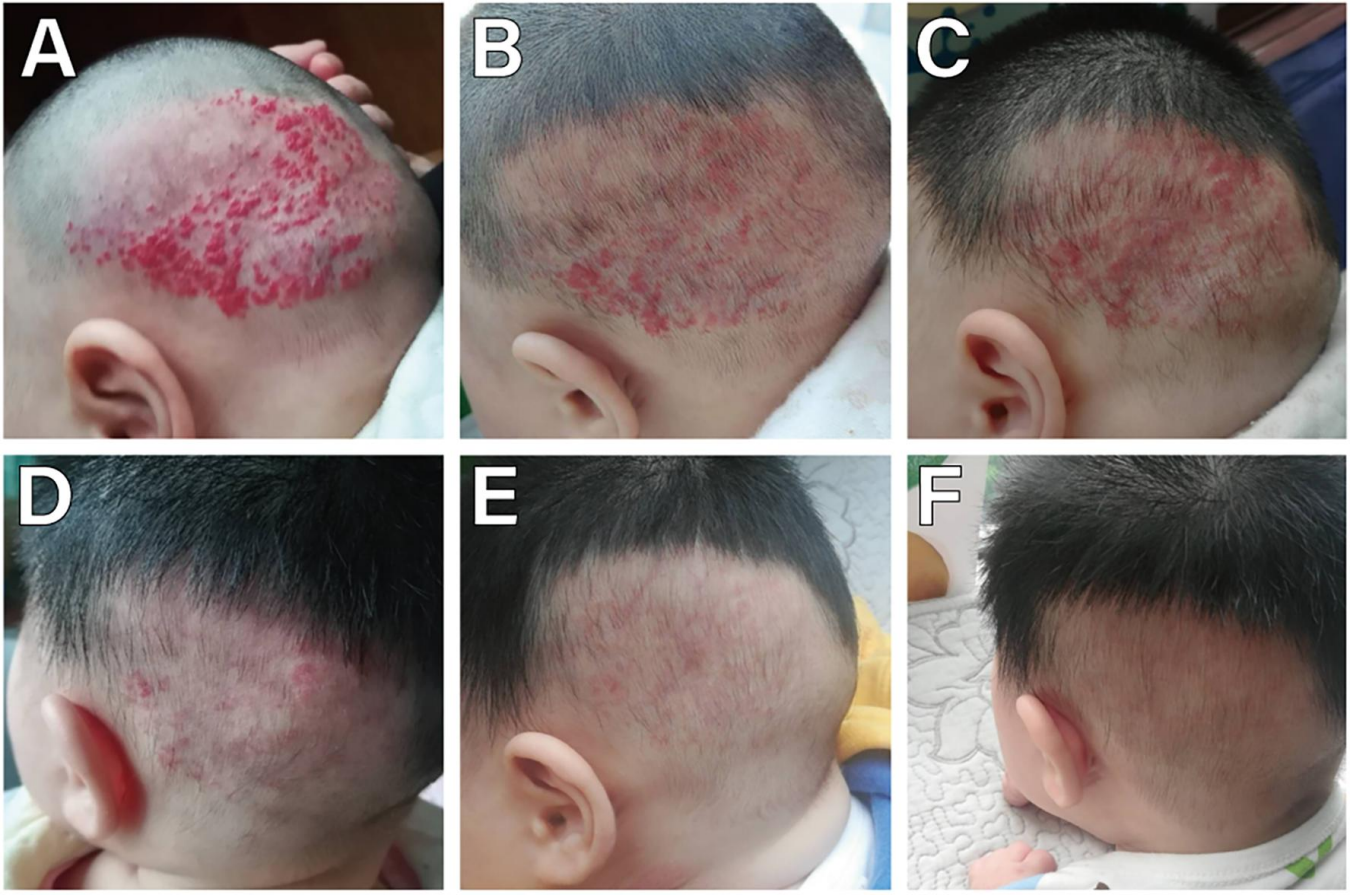
A female with a large superficial IH on the head. **(A)** Before starting topical timolol therapy, at the age of 3 months; **(B)** 1 week after starting topical timolol therapy, the IH shrinked and less red; **(C)** 3 weeks after the initiation of timolol treatment, compared with the 1 week of treatment was not dramaticly; **(D)** 4 weeks after the initiation of timolol treatment, the IH became flatter and less red significantly; **(E)** 5 weeks after the initiation of timolol treatment, IH were almost invisible; **(F)** 2 months after the initiation of timolol treatment, the IH resolved completely.

**Figure 4:**
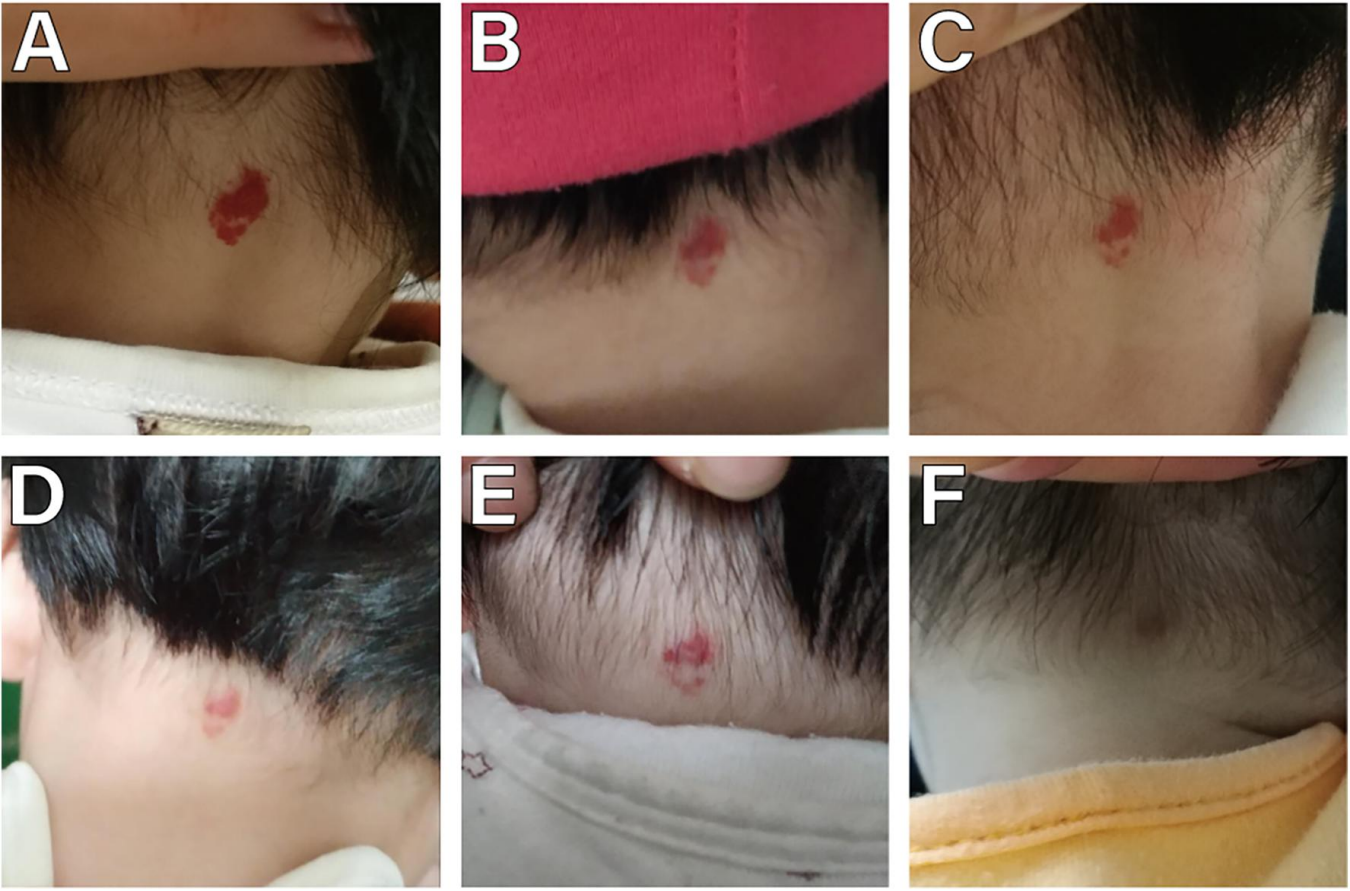
A female with a small superficial IH in the nape. **(A)** Before starting topical timolol therapy, at the age of 4 months and 2 weeks; **(B)** 2 week after starting topical timolol therapy, the IH shrinked and less red significantly; **(C)** 3 weeks after the initiation of timolol treatment, compared with the 1 week of treatment was not dramaticly; **(D)** 4 weeks after the initiation of timolol treatment, IH becomes even less red; **(E)** 5 weeks after the initiation of timolol treatment, IH turned a little red compared with the 4 week of treatment; **(F)** 3 months after the timolol treatment stopped, slight hyperpigmentation was observed.

When using 0.5% timolol maleate eye drops (Wuhan Wujing Pharmaceutical Co., Ltd., National Medicine Standard H42021078), first trim cotton or gauze to match the size of the IH. Apply 3–20 drops to the gauze pad until the gauze is fully saturated but no liquid drips, Apply the wet gauze to the IH surface and seal it with preservative film. During treatment, apply a wet compress three times daily, each lasting 30 min, with an interval of 6–8 h. Monitor the gauze's moisture level throughout. If the gauze dries out, promptly replenish it by dripping additional medicated solution to ensure continuous therapeutic efficacy. The authors confirm that the guardians of the participants have given informed consent for the publication of the images in Figures 1–4.

#### 2.2.2 Medication discontinuation criteria

The completion of the treatment course is determined by meeting either of the following conditions: ① completion of the 12-month standard treatment course (including cases where the lesion has not completely regressed), ② achieving complete IH regression within the 12-month treatment period (permitting early treatment discontinuation). Specifically, for pediatric patients within the 12-month treatment period, a stepwise tapering regimen (reducing daily dosing frequency) could commence after one month of continued medication following complete IH regression without recurrence. Post-treatment follow-up required at least 6 months of observation, with monthly follow-up visits to assess treatment efficacy and potential recurrence.

#### 2.2.3 Follow-up content

All pediatric patients underwent outpatient follow-up after the first received treatment. Follow-up components included: clinical photography, documentation of lesion color and size changes, occurrence of adverse reactions, and assessment of skin sequelae (hyperpigmentation, residual fibrous adipose tissue, scars) and recurrence after discontinuation of medication. Adverse reactions were categorized as local or systemic. Local adverse reactions included skin irritation (itching, desquamation, erythema), erosion, and ulceration. Systemic adverse reactions comprised bradycardia, shortness of breath, asthma, and sleep disturbances.

#### 2.2.4 Evaluation criteria

Treatment outcomes are graded based on changes in IH size. The evaluation was performed by comparison of photographs before and after treatment, measurement of IH diameters with a ruler, and ultrasound examination of the thickness of the IH. Lesion size changes are categorized as Grade 1 “Excellent”, Grade 2 “Good”, Grade 3 “Fair”, or Grade 4 “Poor”. Complete regression or residual microlesions are rated “Excellent” indicating the best prognosis, a reduction of more than half of the original size is judged “Good”, “Fair” indicates a reduction of less than half of the original size, “Poor” denotes no change or progressive enlargement. Treatment outcomes rated “Excellent” or “Good” are classified as positive therapeutic effect, while “Fair” or “Poor” are deemed negative therapeutic effect.

### 2.3 Statistical analysis

Statistical analysis was performed using the SPSS 26.0 statistical software package. Quantitative data are expressed as mean and standard deviation or median and interquartile range. Categorical data are described using numbers and percentages (*N*, %). Independent samples *t*-tests were used to compare quantitative data between two groups. For categorical data, Pearson's chi-square test was employed to compare differences between groups in univariate analysis. If measurement data followed a normal distribution, they were represented as mean ± standard deviation. For non-normally distributed measurement data (or ordinal data), median (25th percentile–75th percentile) was used. Nonparametric Mann–Whitney *U* tests were employed for intergroup comparisons of ordinal data (four levels: Excellent, Good, Fair, Poor). For multivariate analysis of dichotomous data (positive and negative therapeutic effect), binary logistic regression was employed to adjust for confounding factors, yielding corresponding odds ratios (ORs) and 95% confidence intervals (CI). The results were considered statistically significant if *P* < 0.05.

## 3 Results

### 3.1 Clinical features

The age range of 359 children with IH was 1–12 months, with a median age of 2 [interquartile range (IQR), 1–3] months. Among them, 125 were male and 234 were female, yielding a male-to-female ratio of 1:1.872. There were 352 cases of solitary IH and 7 cases of multiple IH. The duration of continuous treatment ranged from 1 to 12 months, with a median treatment duration of 12 (IQR, 12–12) months. The follow-up period ranged from 13 to 29 months, with a median follow-up duration of 21 (IQR, 20–23) months. 263 cases (73.26%) initiated treatment < 3 months (early age) (Figure 2), while 96 cases (26.74%) began treatment ≥3 months (late age) (Figures 1, 3, 4). Among pediatric patients, 147 cases (40.95%) had IH diameters ≤3 cm, and 212 cases (59.05%) had IH diameters > 3 cm (Figure 3). Treatment outcomes were excellent in 117 patients (32.60%), including 52 who achieved complete regression within 12 months of therapy (Figures 1, 3, 4). Good outcomes were observed in 150 patients (41.78%) (Figure 2), fair outcomes in 53 (14.76%), and poor outcomes in 39 (10.86%) (Table 1).

**Table 1:** Demographic and clinical characteristics of patients and infantile hemangioma (IH).

Characteristics	Value
Patients*	359 (100%)
Sex*	
Male	125 (34.82%)
Female	234 (65.18%)
Age(months)**	2 (1–3)
Description*	
Single	352 (98.05%)
Multiple	7 (1.95%)
Treatment duration (months)**	12 (12–12)
Follow-up duration (months)**	21 (20–23)
Age at the beginning of treatment*	
<3 months	263 (73.26%)
≥3 months	96 (26.74%)
Lesion size*	
Small: 0.3 ≤ max diameter ≤ 3 cm	147 (40.95%)
Large: 3 < max diameter < 9 cm	212 (59.05%)
Outcomes*	
Excellent	117 (32.60%)
Good	150 (41.78%)
Fair	53 (14.76%)
Poor	39 (10.86%)
Efficacy evaluation	
Positive therapeutic effect	267 (74.37%)
Negative therapeutic effect	92 (25.63%)

* Values are presented as a number (percentage).

** Values are presented as a median (interquartile range).

### 3.2 Evaluation of treatment outcomes and efficacy

Comparisons among groups with treatment outcomes rated as “excellent, good, fair, poor” revealed no significant association between patient gender and treatment outcome via Mann–Whitney *U* test (*U* = 14,514.5, *Z* = − 0.125, *P* = 0.900). Treatment outcomes were superior in patients aged <3 months (early age) at treatment initiation compared to those aged ≥3 months (late age) (*U* = 9,954, *Z* = − 3.256, *P* = 0.001). and patients in the small size group (0.3 cm ≤ maximum diameter ≤ 3 cm) had better treatment outcomes than those in the large size group (3 cm < maximum diameter < 9 cm) (*U* = 2,869.5, *Z* = − 13.952, *P* < 0.001) (Table 2). Regarding efficacy, univariate analysis employed Pearson's chi-square test. No significant correlation was found between gender and efficacy (*X*^2^ = 0.567, *P* = 0.452). However, significant correlations were observed between efficacy both age at treatment initiation (*X*^2^ = 51.989, *P* < 0.001) and IH size (*X*^2^ = 56.859, *P* < 0.001) (Table 3). After adjusting for multiple confounding factors including gender, age, and IH size, a binary logistic regression analysis was conducted on efficacy groups. Results indicated that age at treatment initiation (OR = 0.784, *P* = 0.024) and IH size (OR = 0.113, *P* < 0.001) (Table 4).

**Table 2:** Infantile hemangioma (IH) patients' outcomes with sex, age and lesion size groups.

Variables	Excellent (*n* = 117)	Good (*n* = 150)	Fair (*n* = 53)	Poor (*n* = 39)	Statistics	*P* Value
Sex (*n*, %)*					*U* = 14,514.5 *Z* = − 0.125	0.900
Male	45 (38.40%)	45 (30.00%)	18 (34.00%)	17 (43.60%)		
Female	72 (61.50%)	105 (70.00%)	35 (66.00%)	22 (56.40%)		
Age at the beginning of treatment (*n*, %)*					*U* = 9,954 *Z* = − 3.256	0.001
<3 months	82 (70.10%)	140 (93.30%)	23 (43.40%)	18 (46.20%)		
≥3 months	35 (29.90%)	10 (6.70%)	30 (56.60%)	21 (53.80%)		
Lesion size (*n*, %)*					*U* = 2,869.5 *Z* = − 13.952	<0.001
Small: 0.3 ≤ max diameter ≤ 3 cm	115 (98.30%)	25 (16.70%)	5 (9.40%)	2 (5.10%)		
Large: 3 < max diameter < 9 cm	2 (1.70%)	125 (83.30%)	48 (90.60%)	37(94.90%)		

* Values are presented as a number (percentage).

**Table 3:** Comparison of demographic and clinical characteristics between patients' efficacy evaluation between well and bad.

Characteristics	Positive therapeutic effect (*n* = 267)	Negative therapeutic effect (*n* = 92)	Statistics	*P* Value
Sex (*n*, %)*			*X*^2^ = 0.567	0.452
Male	90 (33.70%)	35 (38.00%)		
Female	177 (66.30%)	57 (62.00%)		
Age at the beginning of treatment (*n*, %)*			*X*^2^ = 51.989	<0.001
<3 months	222 (83.10%)	41 (44.60%)		
≥3 months	45 (16.90%)	51 (55.40%)		
Lesion size (*n*, %)*			*X*^2^ = 56.859	<0.001
Small: 0.3 ≤ max diameter ≤ 3 cm	140 (52.40%)	7 (7.60%)		
Large: 3 < max diameter < 9 cm	127 (47.60%)	85 (92.40%)		

* Values are presented as a number (percentage).

**Table 4:** Factors predictive of efficacy evaluations evaluation (logistic regression analysis).

Variables	*P*-value	Odds ratio	95% confidence interval
Sex	0.995	1.002	0.491–2.045
Age (month)	0.024	0.784	0.636–0.968
Lesion size (cm)*	<0.001	0.113	0.066–0.195

* Lesion size are presented as a diameter.

### 3.3 Safety analysis

In this study, no cases required discontinuation of treatment due to severe adverse reactions. Only a small number of cases exhibited mild local or systemic adverse reactions. Local adverse reactions occurred in 24 cases (6.69%), while systemic adverse reactions were observed in 41 cases (11.42%). Local adverse reactions primarily manifested as skin irritation (erythema, erosion, ulceration) at the site corresponding to the IH. Systemic adverse reactions mainly included bradycardia, shortness of breath, asthma, and sleep disturbances. Shortness of breath occurred in 27 cases (7.52%), and sleep disturbances in 14 cases (3.90%). No cases of bradycardia or asthma were reported following administration of the medication. Skin sequelae (hyperpigmentation, residual fibrous adipose tissue, scars) were observed in 12 cases (3.34%) (Figure 4F) (Table 5).

**Table 5:** Adverse events with topical timolol treatment.

Adverse events	Value (%)
Local adverse reactions(erythema, erosion, ulceration)	24 (6.69%)
Systemic adverse reactions	41 (11.42%)
Sleep disturbances	14 (3.90%)
Shortness of breath	27 (7.52%)
Asthma	0
Bradycardia	0
Skin sequelae (hyperpigmentation, residual fibrous adipose tissue, scars)	12 (3.34%)

## 4 Discussion

*β*-blockers have now become the first-line alternative to glucocorticoids for treating IH (13). Their time-dependent mechanism of action in IH can be divided into three characteristic phases: early vasoconstriction effects mediated by vascular smooth muscle contraction; The intermediate effect involves downregulating the expression of vascular endothelial growth factor (VEGF), basic fibroblast growth factor (bFGF), and matrix metalloproteinase (MMPs), thereby inhibiting angiogenesis and inducing growth arrest; The late and long-term effect is the induction of vascular endothelial cell apoptosis, ultimately leading to tumor structural remodeling and exerting therapeutic effects (21, 22). This study employed 0.5% timolol maleate as a representative non-selective beta-adrenergic receptor blocker, which possesses unique pharmacological properties. Its beta-adrenergic blocking efficacy is 8 times that of propranolol, while exhibiting no endogenous sympathomimetic activity or direct myocardial depressant effects (23). More importantly, systemic absorption is extremely low with topical administration, and it bypasses hepatic metabolism. Compared to the oral bioavailability of propranolol (approximately 30%), the bioavailability of topically applied timolol is significantly higher (19). In the treatment of superficial IH, topical timolol is equivalent to oral propranolol with fewer adverse reactions (24).

Regarding clinical evidence, multiple studies have confirmed the clinical value of topical timolol in treating IH (25–29). A randomized controlled trial demonstrated that the treatment group exhibited significant reduction in IH thickness and improvement in color as early as week 4, with these changes occurring earlier than in the placebo group (30). Another retrospective cohort study incorporating multicenter data (*n* = 731) further confirmed that topical timolol therapy can simultaneously improve tumor volume and color, and demonstrates superior efficacy for superficial IH compared to mixed-type and deep lesions (31), This difference may be related to the anatomical location and drug permeability of lesions of different subtypes. This study requires all pediatric patients to have preservative film applied over the gauze during treatment to prolong the duration of the medication's effect and enhance its penetration into the deep dermal vessels. This study employed topical treatment with timolol, and no recurrence was observed in any child with favorable prognosis. In contrast, previous studies using oral propranolol for facial IH demonstrated a relatively earlier recurrence time window and a significantly increased recurrence rate (32). The low recurrence rate observed in this study may be associated with the relatively prolonged treatment duration. Furthermore, our follow-up period (median 21 months) was significantly longer than that reported in most literature (typically ≤ 12 months) (22), enhancing the reliability of efficacy assessment and the observed complication rates.

When discussing clinical management strategies for IH, it is essential to fully recognize the dual nature of its natural course. Although it exhibits self-limiting characteristics, the cosmetic defects caused before resolution—particularly in aesthetically sensitive areas such as the head and neck—often result in significant psychosocial impact. Research indicates that the head and neck region is the most common site for ulcerative hemangiomas, and superficial IH lesions in this area are more susceptible to trauma, increasing the risk of bleeding (33), this underscores the necessity of early intervention. From a demographic perspective, the median age (2 months) of the pediatric patients in this cohort were consistent with data reported in previous multicenter studies (31). This study demonstrated that children treated before 3 months of age achieved significantly better treatment outcomes than those treated at or after 3 months (*U* = 9,954, *Z* = − 3.256, *P* = 0.001). Binary logistic regression analysis further confirmed that early intervention (OR = 0.784, *P* = 0.024) was an independent factor influencing positive therapeutic effect. Consistent with studies on oral propranolol treatment for IH, the treatment success rate was significantly higher in the case group which IH therapy was initiated before 10 weeks of age compared to the group which treatment began after 10 weeks of age (34). This highlights the importance of early intervention in IH management, aligning closely with the principle of “early and aggressive intervention” recommended by Christine (9). The findings of this study align with the “therapeutic time window” theory proposed by Tollefson (6), and it may be related to the biological characteristics of the proliferative phase of IH, specifically the heightened sensitivity of proliferating vascular endothelial cells to beta-blockers. Moreover, during the rapid proliferation phase, the hypoxic state of vascular endothelial cells in IH enhances the stability of hypoxia-inducible factor-1α (HIF-1α), which subsequently promotes the expression of multiple angiogenic factors, including VEGF and fibroblast growth factor 2 (FGF2) (35). However, propranolol effectively blocks HIF-1α stabilization, thereby preventing excessive vascular proliferation in IH (36).

In this study, IH size significantly influenced treatment outcomes with timolol (*U* = 2,869.5, *Z* = − 13.952, *P* < 0.001). The therapeutic advantage in children with IH diameters ≤ 3 cm was highly statistically significant (OR = 0.113, *P* < 0.001). This is consistent with the findings of Chan (25), who treated small superficial IH lesions with 0.5% timolol maleate gel. This phenomenon may be explained by the following mechanisms: Smaller lesions allow for more thorough drug penetration, Larger lesions are often accompanied by deeper tissue involvement. However, Borkar successfully treated large and complex facial IH using topical timolol eye drops without any adverse effects during treatment (37). Therefore, topical timolol can also be considered in the large area of IH cases in the head and neck region. In this study, the excellent and good treatment outcomes rate for pediatric patients with IH diameters >3 cm still reached 59.9%, indicating that even in cases with larger IH, clinical benefits may be achieved through standardized treatment for select patients. These findings suggest that topical treatment with timolol may serve as an alternative option for specific types of extensive IH in the head and neck region. However, comprehensive evaluation is required based on the specific characteristics of the lesion (31) (such as thickness and blood supply) and individual patient differences.

Following application of timolol, local adverse reactions such as skin irritation (erythema, erosion, ulceration) and acute contact dermatitis (ACD) have been observed (38). ACD primarily manifests as localized dryness and scales around IH. During treatment of superficial IH in the head and neck region at this center, the primary local adverse reaction was skin irritation, occurring in 24 cases (6.69%). Due to the excellent tolerability of topically applied timolol, ACD caused by timolol is often underestimated or misdiagnosed in most instances (39). This study also did not classify the occurrence of local dryness, itching, and skin desquamation (Figure 2C) in pediatric patients as potential local adverse reactions. Therefore, the incidence rate of local adverse reactions in this study may have been underestimated to some extent.

Regarding systemic adverse reactions caused by the pharmacokinetics of timolol, multiple studies have found detectable concentrations of timolol in the serum of pediatric patients following topical application (40). Its blood concentration may be related to the topical medication dosage, the thickness of the IH, and the application site (41–43). Most studies indicate that topical application of timolol is suitable for small, superficial IH lesions, offering both safety and cost-effectiveness (44). Furthermore, the local application of timolol avoids the first-pass hepatic metabolism effect. Even with systemic absorption issues, its use remains relatively safe in patients with small, superficial IH lesions. The systemic adverse reactions monitored during treatment in this study primarily included shortness of breath in 27 cases (7.52%) and sleep disorders in 14 cases (3.90%). No cases of bradycardia or asthma occurred following medication administration. This result indicated relatively high incidence of systemic adverse reactions related to respiration. We speculate that this might be related to the following two reasons: Firstly, IH in the head and neck region, the scalp may be a high-risk area for systemic absorption, attributed to increased absorption via the sebaceous glands of the scalp hair follicles (41). Secondly, the treatment period for the majority of cases in this study overlapped with the COVID-19 pandemic period, which the guardians' vigilance regarding respiratory symptoms has significantly increased. In summary, although theoretical absorption risks exist with topical timolol treatment for superficial IH in the head and neck region, the actual incidence of systemic adverse reactions is low and symptoms are mild, demonstrating reliable clinical safety.

In this study, skin sequelae (hyperpigmentation, residual fibrous adipose tissue, scars) were observed in only 12 cases (3.34%). Bauland study confirmed that untreated superficial IH resulted in a significantly higher incidence of permanent skin changes compared to deep lesions (74% vs. 25%, *P* < 0.001) (8). Furthermore, scar formation reached 97% in cases complicated by infection, ulceration, or bleeding, topical timolol may improve scar appearance by enhancing the migratory capacity of keratinocytes (45, 46). Studies indicate that untreated IH cases exhibit residual fibrofatty skin lesions in 45.8%–66.7% of cases, with facial lesions showing a higher likelihood of significant residual changes (47). Another prospective study on oral propranolol therapy for IH demonstrated a significantly increased incidence of severe or marked skin sequelae in infants who initiated treatment after 3 months of age (48). This aligns with Chang's recommendation that initiating standardized treatment during the rapid growth phase of IH can reduce irreversible skin sequelae (7). It is evident that the early aggressive treatment of superficial IH in the head and neck region with timolol offers dual benefits. On one hand, the significant color fading observed during the initial treatment phase alleviates parental anxiety and reduces the risk of bleeding due to accidental injury causing IH rupture. On the other hand, it also lowers the incidence of various skin sequelae that may occur during the resolution of IH.

Due to a lack of control group and the retrospective design, this study has several limitations. First, there is a risk of selection bias because the analysis only included pediatric patients who completed the prescribed treatment and follow-up. This may have excluded cases lost to follow-up due to treatment failure and subsequent switching to oral medications or other interventions or surgeries, potentially overestimating the treatment's efficacy and safety. Second, information bias is difficult to avoid, as efficacy and safety assessments rely on historical medical records whose completeness and consistency may be compromised. During the follow-up period, the data and information provided by guardians were susceptible to subjective influence, and the angle and light of the photos taken were inconsistent, which was difficult to standardize. Third, this study evaluates treatment outcomes based on changes in tumor size, yet numerous studies (as mentioned earlier) exhibit inconsistent efficacy assessment criteria, resulting in a lack of objective quantitative metrics. This heterogeneity hinders the identification of the specific causes of efficacy discrepancies. A recent prospective study of topical 2% carteolol in the treatment of IH confirmed that dermoscopic monitoring provides valuable insights into treatment progression, showing the presence of dotted vessels at baseline may serve as a predictor of a favorable treatment response (49). This suggests that future studies should consider incorporating dermoscopic monitoring as one of the metrics for evaluating IH treatment efficacy, thereby further enhancing the evidence level. Fourth, given the lack of timolol serum concentration monitoring in participants, the aforementioned safety analysis only based on reasonable speculation of clinical phenomena by our team and fails to directly establish a causal relationship between the two. Finally, as a single-center study, our conclusions require further validation in prospective, multicenter, randomized controlled trials. Despite these limitations, the findings remain of significant reference value.

## 5 Conclusion

This retrospective study confirmed that 0.5% timolol maleate eye drops administered topically had significant clinical efficacy (74.4% excellent/good response rate) and favorable safety characteristics for superficial IH in the head and neck. Early intervention (<3 months of age) and small lesions (≤3 cm) are key factors for achieving positive therapeutic effect. Therefore, topical timolol may serve as a potential first-line treatment for superficial IH in the head and neck region.

## Data Availability

The original contributions presented in the study are included in the article/Supplementary Material, further inquiries can be directed to the corresponding author.
